# Spatiotemporal continuum generation in polariton waveguides

**DOI:** 10.1038/s41377-019-0120-7

**Published:** 2019-01-16

**Authors:** Paul M. Walker, Charles E. Whittaker, Dmitry V. Skryabin, Emiliano Cancellieri, Ben Royall, Maksym Sich, Ian Farrer, David A. Ritchie, Maurice S. Skolnick, Dmitry N. Krizhanovskii

**Affiliations:** 1 Department of Physics and Astronomy, University of Sheffield, Sheffield, S3 7RH UK; 2 Department of Physics, University of Bath, Bath, BA2 7AY UK; 3 ITMO University, Kronverksky Avenue 49, St. Petersburg, 197101 Russia; 4 Department of Physics, Lancaster University, Lancaster, LA1 4YB UK; 5 Department of Electronic and Electrical Engineering, University of Sheffield, Sheffield, S3 7HQ UK; 6 Cavendish Laboratory, University of Cambridge, Cambridge, CB3 0HE UK

**Keywords:** Nonlinear optics, Polaritons, Supercontinuum generation, Ultrafast photonics

## Abstract

We demonstrate the generation of a spatiotemporal optical continuum in a highly nonlinear exciton–polariton waveguide using extremely low excitation powers (2-ps, 100-W peak power pulses) and a submillimeter device suitable for integrated optics applications. We observe contributions from several mechanisms over a range of powers and demonstrate that the strong light–matter coupling significantly modifies the physics involved in all of them. The experimental data are well understood in combination with theoretical modeling. The results are applicable to a wide range of systems with linear coupling between nonlinear oscillators and particularly to emerging polariton devices that incorporate materials, such as gallium nitride and transition metal dichalcogenide monolayers that exhibit large light–matter coupling at room temperature. These open the door to low-power experimental studies of spatiotemporal nonlinear optics in submillimeter waveguide devices.

## Nonlinear optics: polariton waveguides

Miniature exciton-polariton waveguides make it possible to induce a variety of nonlinear optical effects at very low powers. Paul Walker and co-workers from a UK-Russian team fabricated semiconductor waveguides containing 3 InGaAs quantum wells and a GaAs core and an AlGaAs cladding. Picosecond pulses from a Ti:Sapphire laser (2 ps, 100 W peak power) were coupled into the waveguide from above via a gold grating coupler, they then propagated a distance of 100–600 μm along the waveguide prior to being out-coupled into free space by a second grating coupler. The strong light–matter coupling between excitons and photons inside the waveguide structure induced spectral broadening nonlinear effects such as continuum generation, self-phase modulation, modulational instability and the generation of Cherenkov radiation and X waves. The amount of spectral broadening is determined by the strength of the light–matter coupling.

## Introduction

Optical continuum generation is one of the most dramatic phenomena in nonlinear optics and has wide-ranging applications, including spectroscopy, pulse compression, wavelength division multiplexing, and optical frequency metrology^1^. Initially developed in optical fibers, supercontinuum sources have more recently been demonstrated in on-chip waveguides^2,3^, offering the potential of integrated continuum sources. In systems with no transverse confinement, the spatial envelope of an optical pulse is free to evolve in addition to its temporal envelope. This increased dimensionality opens up rich new physics and applications because of the nonlinear coupling of the spatial and temporal degrees of freedom^4,5^. Spatiotemporal solitons and spectral broadening have been studied in *χ*^(2)^ media^6^ and in arrays of coupled waveguides^7^. Generating significant spectral broadening requires either ~1-cm-long devices or high peak fields, which is usually achieved using broadband, high peak power (~100 fs, megawatt) pulses and/or photonic nanowires with tight transversal confinement, which preclude spatiotemporal effects. Using megawatt-to-gigawatt pulses, optical filamentation^8^ has been observed. This filamentation is accompanied by conical emission^9^, where a cone of radiation surrounds the central core wave packet with different wavelengths appearing at different angles. These rather universal effects have been observed in many systems, including condensed media^10^, air^9^, and water^11–13^.

Nonlinear effects can be exploited at much lower powers using exciton–polaritons. These are hybrid quasi-particles formed by the strong coupling of photons to excitons^14^. In the strong coupling regime, photons coherently exchange energy with electronic excitations of the semiconductor medium, excitons, on a timescale faster than any loss or decoherence mechanisms. New quantum eigenmodes of the system, the lower- and upper-branch polaritons, are formed. They inherit characteristics from each of their constituents. They propagate ballistically like photons, while from their excitonic component, they inherit an effective nonlinearity that is >1000 times^15^ larger than in weakly coupled semiconductors. The high nonlinearity makes the polariton system a candidate for implementing low-power on-chip quantum light sources^16^, all-optical information processors^17^, or nonlinear photonic simulators^18^ of, e.g., topological^19^ or parity-time-symmetric^20^ Hamiltonians.

Traditionally, polariton experiments have been performed using Fabry–Perot microcavities in which the photon field is confined close to the quantum wells between two distributed Bragg reflectors. In such structures, the strong interactions and low polariton mass have allowed the demonstration of condensation^21^, quantized vortices^22^, superfluidity^23^, and solitons^24^. More recently, waveguide polaritons, where the quantum wells couple to the guided or evanescent field of a waveguide, were theoretically^25^ and experimentally^26^ introduced. These polaritons have the advantages of an order of magnitude faster pulse propagation, a stronger photon–exciton coupling, and much less demanding growth and fabrication requirements. Both bright^15^ and dark^27^ solitons have been experimentally observed in polariton waveguides.

The ratio of excitonic to photonic character of a polariton is adjustable by tuning the frequency relative to the exciton resonance over the frequency scale of the photon–exciton coupling, *Ω*, known as the vacuum Rabi splitting, which is usually 3–10 milli-electron volts (meV) in gallium arsenide (GaAs)-based systems. The nonlinearity arises because on-site repulsive interactions shift the underlying exciton resonance to higher frequencies^28^ and hence change the polariton frequency/wavenumber dispersion relation (see Methods and materials—Waveguide nonlinear properties). The strength of the nonlinearity is strongly dependent on the exciton fraction and hence on the frequency detuning from the resonance. This strong spectral inhomogeneity is a key difference from Kerr nonlinear systems. Another important difference is that the nonlinearity for lower polaritons is inherently saturable. As the density increases and the repulsive interactions between excitons shift the exciton resonance to higher frequency, the polaritons at a fixed frequency on the lower branch become more and more detuned from the resonance. Thus they become more photonic in character and less sensitive to further increases in density. Therefore, the strength of the polariton nonlinearity gradually decreases with increasing density.

Since polaritons partially consist of real electronic excitations, a rich new range of interactions opens up compared to photons in dielectric waveguides, including, for example, couplings to phonons, quantum-well disorder potential, or bi-excitons. Such couplings are known to cause scattering of polaritons into a long-lived reservoir of nonradiative excitons, which can then interact with the polaritons and contribute to significantly enhanced nonlinear processes^27,29^. However, the timescale for reservoir build-up is ≥50 ps in GaAs-based samples; thus, it is not relevant to the picosecond experiments we will discuss in this paper. Excitons, and hence polaritons, are also sensitive to magnetic^30,31^ fields, opening up routes to polariton spintronics, and electric fields^32^. Although the polariton nonlinearity we exploit in this paper is already at least 1000 times larger than in weakly coupled semiconductors, it can be enhanced by a further two orders of magnitude by applying electric fields to produce dipolar polaritons^32^. This enhancement may allow nonlinear effects such as the ones that we study in this paper at even shorter length scales and lower powers.

In addition to the nonlinear properties, the strong coupling also has a major impact on the polariton dispersion compared with that of the underlying photon modes. The anti-crossing of the photon and exciton modes over the narrow spectral range, *Ω*, causes the lower (upper) polariton branch to exhibit a giant normal (anomalous) dispersion and, importantly, strong higher-order (than second) dispersions that can dramatically alter pulse propagation phenomena^33^. In the case of microcavities, which have an energy minimum at zero wavenumber, this leads to a lower polariton branch (LPB) with both normal and anomalous regions in a narrow spectral range^24^. This unusual dispersion has been exploited to demonstrate optical parametric oscillation^34^, thermal relaxation from solitonic to condensate-like states^35^, and backward-propagating Cherenkov radiation (CR) in one-dimensional microwires^36^. In both microcavities and waveguides, dispersion and diffraction can have opposite signs so that the system supports spatiotemporal X-waves^37^ and exhibits an unusual curved X-shaped modulation instability gain spectrum in the frequency/angle space^4^, which we demonstrate later. The giant dispersion of polariton systems allows the characteristic dispersion and diffraction length scales to be equal for convenient picosecond pulses and ~5-μm beam widths, which are ideal for on-chip spatiotemporal optics^15^. This is difficult to achieve in conventional photonic systems.

To date, many features of pulse propagation in these systems, especially in the spatiotemporal regime, have remained unclear, particularly the effects of the high-order nonlinear and dispersive terms, which follow from the resonant nature of light–matter coupling. In this work, we study the breakup of and radiation by low peak power (sub-100-W) 2-ps pulses into a spatiotemporal continuum on a submilliliter length scale suitable for integration of on-chip nonlinear devices. The continuum is generated simultaneously in two dimensions: frequency (wavelength) and angular frequency (wavenumber transverse to the propagation direction). This is also the first demonstration of coherent nonlinear optical continuum generation in a polariton system, which is distinct from the many examples of broad spectra generated by incoherent thermal relaxation of polaritons, as in, e.g., ref. ^32^.

We study several regimes corresponding to different input powers. At low powers, spectral broadening is due to self-phase modulation (SPM) caused by polariton–polariton interactions. The spectra in this range are quantitatively reproduced by numerical solution of the coupled photon and nonlinear exciton field equations. We show numerically that the SPM is accompanied by splitting of the input pulse into a train of shorter pulses under the influence of strong spectrally inhomogeneous dispersion and nonlinearity, which arise due to the polaritonic nature of the system. At intermediate powers, a novel form of spatiotemporal modulation instability (STMI)^4,38^ contributes additional spectral broadening, whereas at the highest powers, CR^39^ and X-wave formation become the dominant mechanisms. These processes are analogous to the conical emission from femtosecond filaments.

We are here able to study such phenomena using orders of magnitude less power and shorter devices than the megawatt-to-gigawatt powers and cm-long devices^8^ in which they have been previously observed. We also demonstrate the strong modifications that the light–matter coupling makes to all the processes involved in addition to increasing the nonlinearity. We achieved a total spectral broadening of up to 30 meV at −20 dB points (see Methods and materials—Experiment), which corresponds to 11 times the initial spectral width or >3 times the Rabi splitting of 9 meV with the latter controlling the ultimate limit. We studied a GaAs-based device operating at a temperature of 10 K because this best allowed us to access the fundamental physics involved. Our results may be directly applied to waveguides that are strongly coupled to excitons in other material systems, such as those based on gallium nitride (GaN)^40^ or transition metal dichalcogenides (TMDCs)^41^. In these, the Rabi splitting can be much larger, and these can also operate at room temperature. The polariton waveguide physics we studied is also applicable to other nonlinear systems with strong linear resonances such as media with embedded metal nanoparticles^42,43^.

## Results

### Overview of spectral broadening

**Fig. 1:**
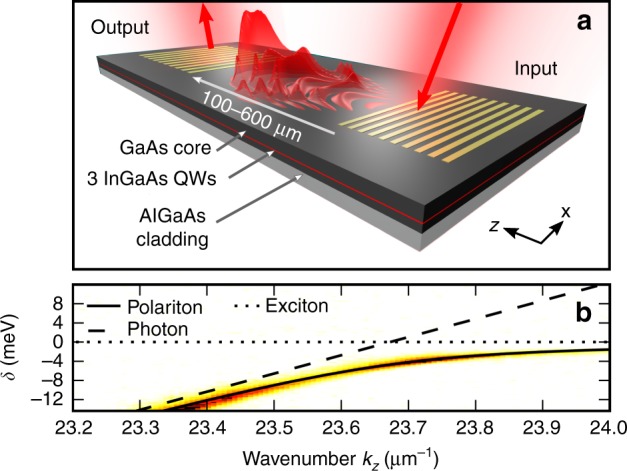
Device properties. **a** Schematic diagram of a waveguide, illustrating light injection and extraction and the propagation of self-phase-modulated pulses. **b** Polariton mode linear dispersion relation shown in angle-resolved photoluminescence with a theoretical fit of coupled photon and exciton modes

**Fig. 2:**
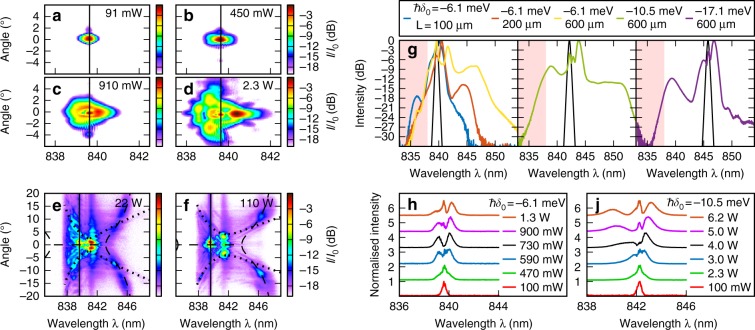
Experimental spectra at the waveguide output. **a**–**d** Spatiotemporal spectra for several input pulse peak powers, *P*, in the low power regime with pump detuning of *δ*_0_ = −6.1 meV and a propagation length of *L* = 600 μm. Solid black lines show the pump wavelength. **e**, **f** Spatiotemporal spectra in the intermediate-to-high power regimes. Dashed and dotted lines show the fitted peak modulation instability gain and Cherenkov radiation phase matching condition, respectively. **g** Comparison of the maximum power spectra integrated over the transverse direction for several *L* and *δ*_0_. The solid black line represents the input spectrum (see Methods and materials—Experiment). The pink shaded region indicates the band of strong linear absorption. **h**, **j** Evolution with power for spectral slices taken through *x* = 0 for two *δ*_0_ and with *L* = 600 μm

Figure 2a–d show the intensity vs. wavelength, *λ*, and angle transverse to the propagation direction of the light at the output. The input pulse central frequency (pump frequency), *ω*_0_, was detuned to ℏ*δ*_0_ = ℏ(*ω*_0_ − *ω*_e_) = −6.1 meV from the exciton resonance at *ω*_e_ (see Fig. 1b). The propagation length was *L* = 600 μm. For the lowest input peak pulse powers, *P*, see Fig. 2a, the spatiotemporal spectrum is narrow in both frequency (wavelength) and angle and corresponds to the spectrum of the input pulses. With increasing power, the spectrum at the output broadens in both frequency and transverse angle, indicating a marked spatiotemporal modulation. At *P* = 2.3 W, the total spectrum broadens by a factor of 3.4 from 2.2 meV to 7.5 meV (at −20 dB points). The broadening is asymmetric with the degree of angular broadening being larger for frequencies closer to the exciton, demonstrating the interdependence of the spatial and temporal degrees of freedom.

At the higher powers shown in Fig. 2e, f, long-wavelength structures appear to form an approximate X-shape centered around the pump frequency. These contribute significant spectral width on the low-frequency side of the generated continuum. For *λ* < 838 nm, light is strongly absorbed in the waveguide by the tail of the inhomogeneously broadened exciton line and does not reach the output coupler. This high-absorption region is typical of polariton systems and is a characteristic of the semiconductor physics underlying the optical properties of polaritons. It arises predominantly from quantum well disorder. Figure 2g compares the spectra at the highest pump powers for a range of *δ*_0_ and *L*. The spectra are integrated over the transverse direction to provide the total intensity vs. wavelength. At the shortest *L* of 100 μm, the spectrum broadens to 13 meV, which is larger than the Rabi splitting, and it increases to 22 meV with increasing *L*. When ℏ*δ*_0_ is moved further from the exciton to −10.5 meV, the width achieved at the highest power increases to 30 meV because less of the generated spectrum is in the high-absorption region. Finally, at ℏ*δ*_0_ = −17.1 meV, the width reduces to 19 meV. This reduction is due to the light–matter interaction being lower for frequencies further from the exciton; thus, for a given power, less nonlinear interaction is possible. Therefore, the strength of light matter coupling ℏ*Ω* = 9 meV controls the limit to achieve spectral broadening.

### Low-power range—SPM by polariton–polariton interactions

We now examine the processes underlying the spectral broadening. Figure 2h, j show sections of the spectra through *x* = 0 over a range of powers and at two different detunings. We first consider Fig. 2h. At the lowest powers, the spectrum is centered on the pump frequency. With increasing power, symmetric wings appear on either side of the central peak and grow to the same height. The central peak then reduces to zero intensity before growing again as the sidebands move further away. This pattern is well known as the signature of SPM, the most fundamental nonlinear process in waveguides or optic fibers^33^. We achieved this modulation at powers and device lengths corresponding to a waveguide nonlinear parameter of 1/(*PL*_NL_) = 16,900 (Wm)^−1^, which is orders of magnitudes more nonlinear than semiconductors with a photon-only nonlinearity^3,44^ (see Methods and materials—Waveguide nonlinear properties). Note the strong asymmetry that develops at higher powers. The sideband on the high-frequency (short-wavelength) side is spread out compared with that on the low-frequency side. This effect is more pronounced for ℏ*δ*_0_ = −10.5 meV, as shown in Fig. 2j. The SPM, and hence the spectral spreading, are stronger for the frequency components closer to the exciton resonance. This is a signature of the polariton–polariton interactions that underlie the nonlinear process. Polaritons at frequencies closer to the exciton resonance have a higher excitonic fraction and thus experience higher nonlinear modulation. This frequency-dependent nonlinearity also explains why the angular broadening discussed earlier is larger for the frequencies closer to the exciton. Thus the triangle-like spectrum in Fig. 2d is a characteristic of pulse self-modulation in polariton systems (see Supplementary Note 1 for more details).

### Comparison of the low-power regime with numerical modeling: self-steepening and pulse splitting

To obtain more insight into the nonlinear processes in this light–matter system, we solved the coupled equations for the photon and nonlinear exciton fields given in Eq. (1).

1a $$\left[ {i\frac{\partial }{{\partial t}} + i\gamma _{\mathrm{p}} + v_g\left( {i\frac{\partial }{{\partial z}} + \frac{1}{{2k_{\mathrm{e}}}}\frac{{\partial ^2}}{{\partial x^2}}} \right)} \right]A = \left( {\frac{{\mathrm{\Omega }}}{2}} \right)\Psi$$

1b $$\left[ {i\frac{\partial }{{\partial t}} + i\gamma _{\mathrm{e}} - g\left| \Psi \right|^2} \right]\Psi = \left( {\frac{{\mathrm{\Omega }}}{2}} \right)A$$

**Fig. 3:**
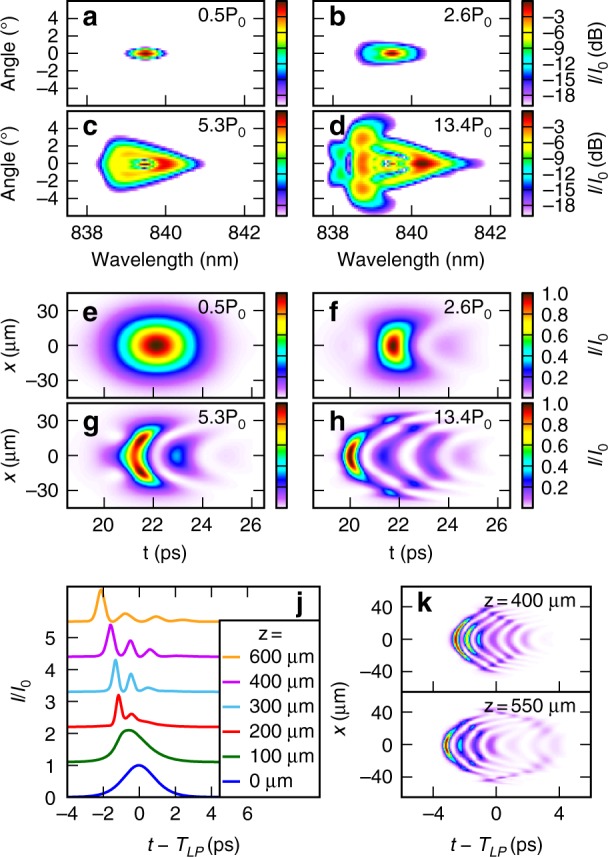
Pulse breakup into a femtosecond pulse train. **a**–**d** Numerical spectra of the photon field at the waveguide output. **e**–**h** Corresponding spatiotemporal envelopes. **j** Sections of the wave packet envelope through *x* = 0 at several *z* for input conditions corresponding to **h**. **k** Spatiotemporal envelopes for two different *z* for *P* = 38.1 *P*_0_. *T*_LP_ is the propagation time over distance *L* using the polariton group velocity at the pump frequency, as defined in “Methods and materials—Waveguide linear properties.” The powers are scaled in units of *P*_0_, which is defined at the end of “Methods and materials—Numerical solution of coupled field equations”

Here, *A* and *Ψ* are the photon and exciton envelope amplitudes, respectively. Then, *v*_g_ and *k*_e_ are the photon group velocity and wavenumber at *ω*_e_, respectively. Then, *γ*_p_ and *γ*_e_ are the photon and exciton loss rates, respectively, and *g* is the exciton frequency renormalization per unit exciton density. The equations were solved numerically using experimentally determined values for the parameters (see Methods and materials—Numerical solution of coupled field equations). Figure 3a–d show the simulated spectra that correspond to the same parameters as the experimental spectra in Fig. 2a–d. Upon comparing Figs. 2a–d and 3a–d, we observed that the modeling reproduces the detail of the experimental spectrum vs. angle and wavelength as it evolves over the entire power range. This finding confirms that the experimental spectral broadening in this power range is due to polariton–polariton interactions since no other semiconductor-specific processes are included in this model.

Figure 3e–h show the wave packet intensity envelopes vs. *x* and *t* corresponding to the spectra in Fig. 3a–d, and Fig. 3j shows sections through *x* = 0 as the wavepacket of Fig. 3d propagates. The changes in the spectrum are accompanied by a reshaping of the pulse envelope in space and time. With increasing power, the pulse at the output breaks into a train of curved wavefronts with a temporal full-width-at-half-maximum (FWHM) of ~450 fs. Experimentally, we cannot measure these subpicosecond temporal dynamics; however, the very good agreement between the experimental and numerical spectra means that we can be confident that the numerical solution accurately describes the nonlinear evolution of the pulse so that we can draw conclusions from the numerical temporal dynamics. The pulse dynamics proceed as follows with further details given in Supplementary Note 1. As the pulse propagates, the SPM creates new high (low) frequency components on the leading (trailing) edge that cause spectral broadening. Since the nonlinearity is stronger for the higher frequencies (closer to the exciton), the broadening is larger on the high frequency side, as shown experimentally in Fig. 2h, j. This leads to steepening of the leading edge, as shown in Fig. 3j at 100 μm propagation. Thus the frequency dependence of the polariton nonlinearity leads to a self-steepening-like effect during the early stages of pulse propagation before pulse breakup. With further propagation, the dispersion, where the leading order is of opposite sign to the nonlinearity, acts on the broadened spectrum to temporally compress the pulse. The spectral inhomogeneity of the system then leads to splitting into several narrow pulses with different central frequencies instead of just one narrow peak. In principle, either the higher-order dispersion terms or the frequency dependence of the nonlinearity, both of which are caused by the strong photon–exciton coupling, may be dominantly responsible for the pulse breakup (see Supplementary Note 1 for more details).

In our previous work^15^, we experimentally showed that quasi-solitonic propagation in this system is possible for wave packets that are spatially extended (>20 μm FWHM) in the transverse direction. The conditions are very similar in this work; thus we may expect the wave packet to propagate as a train of quasi-one-dimensional (quasi-1D) solitons that are spatially extended in the transverse direction along with some background radiation. The pulse breakup may then be viewed as destabilization of a high-order soliton by the spectrally inhomogeneous polariton dispersion or nonlinearity^45^. The essential contribution of the polaritonic nature of the system is that the wave packet modulation may occur on short length scales because of the large magnitude of the dispersion and polariton–polariton interactions brought about by the strong coupling.

The frequency dependence of the nonlinearity also has a strong impact on the spatial dynamics. With increasing propagation (or higher pump power), the wavefronts of the pulse train become increasingly curved, and eventually, the outer edges of the pulse train separate into a secondary wavefront with opposite curvature, which is shown to occur between *z* = 400 and 550 μm in Fig. 3k. The curvature and separation occur because SPM also acts on the spatial envelope to generate new in-plane momentum components, causing the pulse to defocus and spread out in *x*. Since the polariton nonlinearity is frequency dependent, the frequency components close to the exciton experience the strongest defocusing and move to a higher *x*. They are also the slowest propagating frequency components because of the strong dispersion. Thus the components at high *x* lag behind the rest, leading to curved wavefronts and eventual separation of the high-frequency components from the outer trailing edge of the train. This effect arises from both the frequency dependence of the polariton nonlinearity and the strong polariton dispersion.

### Intermediate-to-high-power regime

We now turn our attention to the features observed at intermediate powers shown in Fig. 2e, f. In both figures, we observe an intense core near zero angle and close to the pump wavelength. This is surrounded by spectrally broad emission along contours with the angle increasing as the frequency becomes farther from the exciton. These patterns of radiation surrounding an intense core are analogous to the conical emission that accompanies the SPM-broadened core in filamentation^8^.

First, in Fig. 2e, on the high-frequency (short-wavelength) side of the pump, we see a spectrum with angular width that increases as the frequency approaches the exciton. This is consistent with the observed trend (e.g., Fig. 2d) and is expected for SPM-generated spectra in this system. On the low-frequency side of the pump, however, we also observe clear spectral emission along the contours with the angle increasing as the frequency becomes farther from the exciton, which cannot be explained by the SPM mechanism. In Fig. 2f, the qualitative shape of the spectrum changes once again. The dominant features on the long wavelength side are nearly linear with a curvature only visible at the frequencies farthest from the exciton. Extra emission also becomes clearly visible on the short wavelength side with much greater curvature than that on the long wavelength side. The straight line features on the long wavelength side are already faintly visible at the lower power, as shown in Fig. 2e, where they cross the dominant emission contours at ~8 degrees. This finding suggests that two mechanism compete at these intermediate-to-high powers.

These two patterns of radiation are well explained by the mechanisms of STMI^4,38,46^, CR^39^, and the formation of spatiotemporal X-waves^11,37,47^ with different mechanisms dominating in the intermediate (Fig. 2e) and high (Fig. 2f) power regimes.

In STMI, spontaneous fluctuations on top of a plane-monochromatic pump wave are unstable to exponential growth. The amplification of the fluctuations at frequencies of *δ*_0_ ± *Δ* occurs via a four-wave mixing process that is nonlinearly phase matched by the SPM^33^. This mechanism has been successful in explaining conical emission patterns despite the pump being pulsed rather than a true plane-monochromatic wave^8^. The CR and X-wave mechanisms are closely related^12^. CR occurs when the straight line dispersion of a quasi-soliton or nonlinear wave packet crosses the dispersion relation of the linear waves of a waveguide. Then the soliton may efficiently radiate power into the dispersive waves because the two stay in phase over a significant propagation length. This phase matching selects linear waves that lie close to the straight line dispersion of the soliton. X-waves are linear, nonspreading wave packets that occupy a region in (*k*_*x*_, *δ*) space such that the longitudinal and transverse dispersions cancel and all components travel at the same velocity, e.g., on a straight-line dispersion curve very much like that of solitons. Thus they follow the same *k*_*x*_(*δ*) dependence as expected for Cherenkov emission.

In the following sections, we compare the experimental data with theoretical calculations of the spectral patterns expected for these two mechanisms.

### Comparison of the experiment with the STMI process

We calculated the gain spectrum of STMI from monochromatic plane waves centered at the input pulse central frequency (see Methods and materials—Spatiotemporal modulation instability). There are two essential features. First, for frequencies close to the pump, ${\mathrm{\Delta }}^2 \le {\mathrm{\Delta }}_s^2$, the peak gain occurs at *k*_*x*_ = 0, whereas it occurs at a finite *k*_*x*_ for frequencies farther from the pump. Second, the gain is cutoff for large *Δ*.

**Fig. 4:**
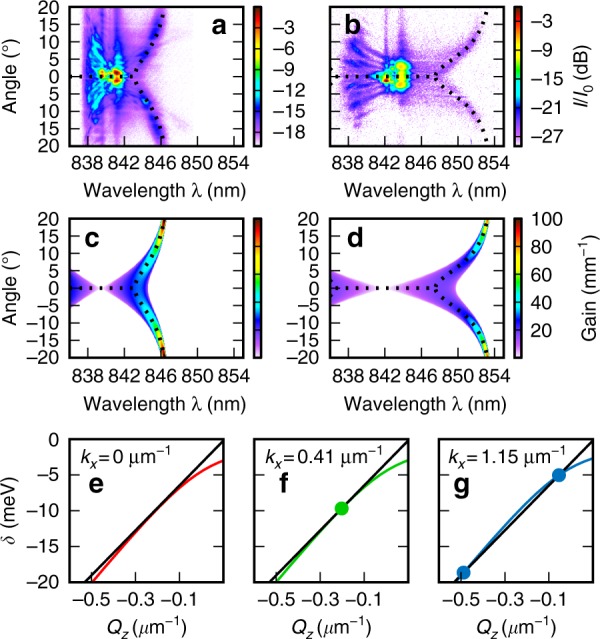
Modulational instability and Cherenkov radiation. **a**, **b** Experimental spatiotemporal spectra at high power for two different initial pulse detunings. **c**, **d** Calculated modulational instability gain spectra for pump frequencies corresponding to **a**, **b**. **e**–**g** Illustration of the Cherenkov radiation phase matching condition. The black line shows the straight line (soliton) dispersion, and the colored lines are the linear polariton dispersion vs. longitudinal wavenumber for three different transverse wavenumbers, *k*_*x*_. Circles indicate the crossing points, which have none in **e**, one in **f**, and two in **g**

Figure 4a, b show the experimental spectra at the maximum power for two *δ*_0_. The bright central part of the spectra near *θ* = 0 and the pump wavelength are due to the SPM-related effects that were discussed earlier. Figure 4c, d show the calculated STMI gain spectrum. The dotted lines provide the transverse angle at which the gain is maximum for each wavelength. The same dotted lines are plotted on Fig. 4a, d. The *θ*(*λ*) dependence of the features, the wavelength where they meet at zero angle, and the cutoff frequency all simultaneously agree with the curvature, the critical frequency Δ_*s*_, and the cutoff of the theoretical peak gain curves. This is the case for both pump frequencies. This is strong evidence that they originate from the STMI mechanism. Of note, the corresponding emission on the short wavelength side of the pump could not be observed because it is in the strong absorption band of the exciton.

### Comparison of the experiment with the Cherenkov/X-wave emission process

The phase matching condition for these processes is shown graphically in Fig. 4e–g with the mathematical form given in Methods and materials—Phase matching condition for CR/spontaneous X-wave formation. The spatiotemporal frequencies at which the condition is fulfilled are plotted as dotted lines in Fig. 2f for straight line dispersion parameters of *v*_s_ = 47.7 μm ps^−1^ and *Q*_s_ = 0.106 μm^−1^, which were obtained from a best fit of the solutions of Eq. (5) to the experimental data. Good agreement was obtained over a wide range of frequencies, indicating that the radiation is indeed due to the selection of linear dispersive waves with a single velocity, which is consistent with CR. Irrespective of the particular parameters used, this mechanism provides the correct qualitative features of emission on both sides of the pump with low curvature on the long-wavelength side and increasing curvature as the frequency approaches the exciton. At this highest power, it is likely that there is a complicated interplay of SPM, modulational instability (MI), soliton dynamics, and CR/X-wave formation.

### Effect of strong coupling at the intermediate-to-high-power regime

As discussed earlier, the strength of the polariton–polariton interaction makes these spatiotemporal effects observable with unprecedented short length scales and low power pulses. The polaritonic nature of our system also introduces a number of qualitative differences compared with weakly coupled systems. The strong peak in the STMI spectrum at nonzero frequency detuning *Δ* arises from the resonances in the phase matching introduced by the light–matter coupling, as has been theoretically predicted in the 1D (temporal only) case^46^. Furthermore, the asymptotic approach of the peak gain toward a maximum frequency at high angles is caused by the strong high-order dispersion terms^38^ that arise from the anti-crossing due to the strong photon–exciton coupling. This is qualitatively different from conventional STMI^4^. The polaritonic dispersion is also responsible for the unconventional highly curved Cherenkov emission pattern in the angle-frequency plane.

## Discussion

In conclusion, we have studied spatiotemporal evolution of pulses in a planar polariton waveguide. We have experimentally observed SPM, MI, and CR/spontaneous X-wave formation, all of which are strongly modified by the resonant nature of light–matter coupling. The very strong polariton nonlinearity allows generation of a continuum that is up to 4 Rabi splittings wide and observation of spatiotemporal effects that are analogous to conical emission at unprecedented low-power densities and propagation lengths. These could be made even lower in future works by exploiting dipolar polaritons^32^. The results compare well with theoretical modeling based on the coupled equations for photon and exciton fields. We found a quasi-self-steepening mechanism arising from the frequency dependence of the polaritonic nonlinearity. We deduced that high-energy input pulses become split into a train of shorter pulses over a short propagation length because of the strong polariton dispersion and nonlinearity. Then they form a wave train with characteristic curved wavefronts in which the high frequency components move to the outer edges and are shed. The space–time coupling offers the potential for on-chip spectral or pulse-train sources that may be tuned simply using the angle of propagation. Although the spectral width is limited by the Rabi splitting, it may be increased using materials with higher exciton oscillator strengths and binding energies, such as those based on GaN^40^ or TMDCs^41^, and/or by tailoring the dispersive properties of the waveguide similar to photonic crystal fibers. The many possibilities for active materials also make a wide range of central wavelengths possible.

## Methods and materials

### Experiment

The sample was excited by a mode-locked titanium-doped sapphire laser emitting picosecond pulses at a repetition rate of 80 MHz. The spectral and autocorrelation FWHM of 0.70 ± 0.05 meV and 3 ps are consistent with 1.9 ± 0.1 ps FWHM sech^2^ pulses. The spectral width includes the instrument response of our spectrometer, which was measured to be approximately 0.05 meV using a single-mode laser. The maximum average beam power incident on the sample was 117 mW, corresponding to a peak pulse power of 733 W. The light was coupled to the sample via a diffractive grating coupler fabricated from thermally evaporated gold using electron beam lithography and a lift-off process. The incidence angle, laser polarization, and incidence position relative to the edge of the grating were tuned to maximize the coupling to the guided mode by maximizing the emission observed at the output coupler for a low incident power. We estimate a coupling loss of 8.2 dB (15% coupled) based on finite-difference-time-domain calculations, giving a maximum coupled peak pulse power of 110 W. The spot size incident on the coupler was a diameter of 37 μm; thus the peak power density per unit transverse width was <3 W μm^−1^. The light was collected by a microscope objective, and either the near or far field was imaged onto the input slit of an imaging spectrometer and recorded by a charge-coupled device (CCD) camera. Thus the spectra were recorded as a function of *x* and *λ* or *θ* and *λ*. To compare total spectral widths, the CCD images were integrated in the transverse direction to provide total intensity vs. *λ*. Following the review by Dudley et al.^1^, we choose the −20-dB point for the measurement of the spectral width. At this point, the intensity is 1% of the value at the peak and more than an order of magnitude above the noise floor for the high power spectra shown in Fig. 2g. To display the input spectra in Fig. 2g, the experimental data were fit with a sech^2^ profile, and the fit is plotted on the graph. This is because, at the low powers for which nonlinear effects are negligible, the noise floor is at −20 dB. While this is sufficient to obtain a good fit, it would make the figure look untidy if the raw data were plotted. The photoluminescence spectrum in Fig. 1 was measured by exciting a piece of the same wafer using a laser at a frequency above the band gap of the quantum wells. Hot carrier relaxation ensures low density occupation of all the polariton modes. The emission was collected from a grating near to the excitation spot in two linear polarizations that corresponded to the transverse-electric (TE) and transverse-magnetic (TM) linearly polarized modes of the waveguide. These spectra were subtracted to remove the residual unpolarized emission at the exciton frequency, leaving only the TE-polarized emission due to the polaritons.

### Waveguide linear properties

Equations (1a, b) describe the evolution of the coupled linear photon and nonlinear exciton fields. The envelope fields, *A* and *Ψ*, are taken to vary slowly around exp(*ik*_e_*z* − *iω*_e_*t*). The photon mode is taken to be dispersionless, which is described only by its group velocity, *v*_g_, and wavenumber, *k*_e_, at exciton frequency, *ω*_e_, since the photonic dispersion is negligible compared with that caused by the coupling to the exciton. Neglecting nonlinearity and loss, Eq. (1) can be solved to obtain the polariton dispersion relation. The longitudinal component, *Q*_*z*_, of the wave vector is given by Eq. (2a).

2a $$Q_z = k_z - k_{\mathrm{e}} = \frac{1}{{v_{\mathrm{g}}}}\left[ {\delta - \frac{{\left( {{\mathrm{\Omega }}/2} \right)^2}}{\delta }} \right] - \frac{{k_x^2}}{{2k_{\mathrm{e}}}}$$

2b $$L_{\rm {loss}} = v_{\rm{g}}/\left[ {2\gamma_{\rm{p}} + 2\gamma_{\rm{e}}\Omega ^2/(2d)^2} \right]$$

Here *δ* = *ω* − *ω*_e_, and *k*_*x*_ is the transverse component of the wave vector. The second-order dispersion, ∂^2^*Q*_*z*_/∂*ω*^2^, is positive for all frequencies on the LPB (*δ* < 0), corresponding to so-called normal group velocity dispersion. The linear losses arise from both photonic losses (e.g., absorption in bulk GaAs and tunneling through the lower cladding) and loss from the exciton component due to, e.g., scattering by phonons. The linear loss length is well described by Eq. (2b) for most frequencies; however, for those above *δ* = −3.5 meV, there is additional strong loss that can be viewed as the frequency-dependent absorption of photons arising from the inhomogeneous broadening of the exciton line.

Equation (2a) can be used to obtain the group velocity, $v_{{\mathrm{g,pol}}} = v_{\mathrm{g}}\delta _0^2/\left( {\delta _0^2 + \left( {{\mathrm{\Omega }}/2} \right)^2} \right)$, of a polariton pulse with central frequency *δ*_0_. The pulse propagation time over distance *L* (in the linear regime, e.g., at low power) may then be obtained as *T*_LP_ = *L*/*v*_g,pol_.

### Waveguide nonlinear properties

Here we give a qualitative picture of the origin of the resonant polariton nonlinearity. The nonlinear term in Eq. (1b) induces a blueshift of *ω*_NL_ = *g*|*Ψ*|^2^ for the exciton resonance proportional to the density of excitons, |*Ψ*|^2^. This causes a renormalization of the polariton dispersion relation so that the longitudinal wavenumber changes by the (frequency-dependent) amount *Q*_NL_ given in Eq. (3), where *δ*_*R*_ = *δ* − *ω*_NL_. This is a lowest-order approximation, which is valid for monochromatic waves or for pulses of low power or propagating over a short propagation length such that the change in intensity envelope due to the intrinsic self-steepening effect can be ignored.

3 $$Q_{{\mathrm{NL}}} = \frac{{\omega _{{\mathrm{NL}}}}}{{v_{\mathrm{g}}}}\frac{{\left( {{\mathrm{\Omega }}/2} \right)^2}}{{\delta \delta _R}}$$

The intensity-dependent change in wavenumber leads to the accumulation of an intensity-dependent nonlinear phase, *ϕ*_NL_(*I*) = *Q*_NL_(*I*)*L*, during propagation. The intensity and hence the phase are functions of *t* and *x*; thus the pulse becomes chirped, which is the origin of SPM. The origin of new frequency components can be observed by considering the instantaneous frequency, *ω*_*i*_(*t*) = ∂*ϕ*(*t*)/∂*t*. The shape of the spectrum is characteristic of the phase shift, *ϕ*_pk_, at the peak intensity. The equivalent chirp in the *x* direction combined with diffraction leads to nonlinear spatial defocusing. The evolution of the SPM spectrum with power may be used to deduce the size of the nonlinearity. In a real system, we must account for loss. In this case, the nonlinear phase develops over an effective propagation length^33^ of *L*_eff_ = *L*_loss_[1 − exp(−*L*/*L*_loss_)], which is less than *L*. Considering Fig. 2h and the lowest powers where the spectrum is still symmetric, we achieve *ϕ*_pk_ = 1 for a peak power of *P* = 174 mW with *L*_eff_ = 340 μm (*L* = 600 μm; *L*_loss_ = 480 μm). The nonlinear length, *L*_NL_ = *L*_eff_/*ϕ*_pk_, is then 340 μm, and the waveguide nonlinear parameter is *γ* = 1/(*PL*_NL_) = 16,900 (Wm)^−1^ for the transverse width of 37 μm, which is consistent with ref. ^32^. We note that *γ* can be increased by reducing either the transverse width or the detuning *δ*_0_. In comparison, in semiconductors with a photon-only nonlinearity, *ϕ*_pk_ = 1 requires devices an order of magnitude longer, narrower, and/or with a higher power, for example, *P* = 22 W in a 9-mm-long aluminum-gallium-arsenide (AlGaAs)^44^ device with a width of 3.5 μm or ~140 mW in a 10-mm-long, 0.5-μm-wide silicon device^3^.

### Numerical solution for the coupled field equations

Equations (1a, b) were solved in the (spatial) Fourier domain and integrated with *t* using an exponential time differencing (ETDRK4) scheme^48^. The nonlinear terms were evaluated in the real-space domain using a Fourier transform pair. All input parameters were taken from experimental measurements. The values used were *γ*_p_ = 33 μeV, *γ*_e_ = 13 μeV, *v*_g_ = 58 μm ps^−1^, *k*_e_ = 23.7 μm^−1^, and *Ω* = 9 meV. A photonic pump term, $\sqrt {P\left( {x,z,t} \right)} \,{\mathrm{exp}}\left[ {i\left( {Q_0z - \delta _0t} \right)} \right]$, was added to the right hand side of Eq. (1a), where the pump intensity, *P*, is real and follows a sech^2^ temporal profile with a 2-ps FWHM and a 37-μm FWHM Gaussian spatial profile. The value of *Q*_0_ was obtained from Eq. (2a) with *k*_*x*_ = 0 and *δ* = *δ*_0_.

The input pulse powers were scaled in units of *P*_0_, which is defined as follows. In the limit as *α* → 0, a pulse with peak power *αP*_0_ will acquire a nonlinear self-phase of *α* when it propagates over the length of the device. Quantitatively, we have $P_0 = 1/( {L_{{\mathrm{eff}}} \cdot \mathop {{\lim }}\limits_{P \to 0} \partial Q_{{\mathrm{NL}}}/\partial P} )$. Here *P* is the pulse power in simulation units, and *Q*_NL_ is the nonlinear change in the polariton propagation constant at power *P*, which is obtained from the simulation via Eq. (3) using peak |*Ψ*|^2^ at *z* = 0 obtained from the simulation output. *L*_eff_ is the effective device length, taking into account loss. For more details regarding *Q*_NL_ and *L*_eff_, see “Methods and materials—Waveguide nonlinear properties.”

### Spatiotemporal modulation instability

STMI occurs via a four-wave mixing process from the pump to signal and idler waves with frequencies and wavevectors detuned by ±*Δ* and $\pm \left( {k_x\widehat {\boldsymbol{x}} + q\widehat {\boldsymbol{z}}} \right)$, respectively, from the pump, where *Δ* is positive. The STMI gain, *G*, is given in Eq. (4a) (for a detailed derivation, see Supplementary Note 2).

4a $$G = \frac{2}{{v_{\mathrm{g}}}}\left[ {{\mathrm{\Delta }}^2\eta \left( {\eta _0 - \eta } \right) - 2\eta \left( {\frac{{{\mathrm{\Delta }}^2}}{{\delta _{\mathrm{R}}}} + \omega _{{\mathrm{NL}}}} \right)\omega _{\mathrm{T}} - \omega _{\mathrm{T}}^2} \right]^{1/2}$$

4b $$\eta = \frac{{\left( {{\mathrm{\Omega }}/2} \right)^2}}{{\left( {\delta _{\mathrm{R}} - {\mathrm{\Delta }}} \right)\left( {\delta _{\mathrm{R}} + {\mathrm{\Delta }}} \right) - 2\omega _{{\mathrm{NL}}}\delta _{\mathrm{R}}}}$$

4c $$\eta _0 = \left( {{\mathrm{\Omega }}/2} \right)^2/\delta _{\mathrm{R}}^2$$

4d $$k_{\mathrm{m}} = \left[ { - \frac{{2k_{\mathrm{e}}}}{{v_{\mathrm{g}}}}\frac{\eta }{{\delta _{\mathrm{R}}}}\left( {{\mathrm{\Delta }}^2 - {\mathrm{\Delta }}_{\mathrm{s}}^2} \right)} \right]^{1/2}$$

Here, *ω*_NL_ is the nonlinear renormalization of the exciton frequency due to the occupation of the pump state, and *δ*_R_ = *δ* − *ω*_NL_ is the corresponding renormalized pump-exciton frequency detuning. The physical interpretation of $\omega _{\mathrm{T}} = v_{\mathrm{g}}k_x^2/\left( {2k_{\mathrm{e}}} \right)$ is the kinetic energy associated with transverse wavenumber *k*_*x*_ relative to the pump. Meanwhile, *η*_0_ is the ratio of the exciton to photon densities in the pump, and *η* is the same but averaged over the pump signal and idler. For a detuning that is less than some critical value of ${\mathrm{\Delta }}^2 \le {\mathrm{\Delta }}_{\mathrm{s}}^2 = - \delta _{\mathrm{R}}\omega _{{\mathrm{NL}}}$, the peak gain occurs at *k*_*x*_ = 0, e.g., for waves travelling with the same transverse momentum as the pump. For ${\mathrm{\Delta }}^2 > {\mathrm{\Delta }}_{\mathrm{s}}^2$, the peak gain occurs at a finite *k*_*x*_ = *k*_m_ given in Eq. (4d). The gain cutoff at high Δ occurs for ${\mathrm{\Delta }}^2 > \delta _{\mathrm{R}}^2 - 2\delta _{\mathrm{R}}\omega _{{\mathrm{NL}}}$. For the comparison with experimental data in Fig. 4, the value of *ω*_NL_ was treated as a fitting parameter. We obtained ℏ*ω*_NL_ = 4.0 and 5.7 meV for ℏ*δ*_0_ = −6.1 and −10.5 meV, respectively. The corresponding nonlinear lengths of 29 and 56 μm may be estimated using Eq. (3) and are consistent with the nonlinear lengths of the ~350 fs fundamental solitons previously observed for this system^15^.

### Phase matching condition for CR/spontaneous X-wave formation

Here we consider the phase matching of the linear waves of the system, with the dispersion relation given by Eq. (2a), with the straight line dispersion relation, *δ* = *v*_s_(*Q*_*z*_ − *Q*_s_), where *Q*_s_ is a constant. This dispersion has a constant (frequency-independent) velocity, *v*_s_ and crosses the exciton frequency at wavenumber *k*_e_ + *Q*_s_. This dispersion relation can describe the quasi-1D solitons previously observed for this system^15^. Taking the two dispersion relations and eliminating *Q*_*z*_ gives Eq. (5) for frequencies *δ* at which the two cross. Here *σ*^2^ = *v*_s_/(*v*_g_ − *v*_s_) and $Q_1 = Q_{\mathrm{s}} + k_x^2/\left( {2k_{\mathrm{e}}} \right)$.

5 $$\delta = - \frac{{v_{\mathrm{g}}Q_1}}{2}\left[ {\sigma ^2 \pm \sqrt {\sigma ^4 - \sigma ^2\left( {\frac{{\mathrm{\Omega }}}{{v_{\mathrm{g}}Q_1}}} \right)} } \right]$$

Equation (5) provides the frequencies, *δ*, as a function of *k*_*x*_, for which CR is efficient. It also provides the frequencies as a function of *k*_*x*_ for the linear waves that all travel together at velocity *v*_s_, e.g., those that can contribute to a spatiotemporal X-wave with a velocity of *v*_s_.

## Supplementary information

Supplementary Note 1: Pulse Dynamics

Supplementary Note 2: Modulation Instability
