# Supplementary material for: Spatiotemporal continuum generation in polariton waveguides
Source: Light Sci Appl. 2019 Jan 16;8:6. doi: 10.1038/s41377-019-0120-7 (PMC6333623; doi:10.1038/s41377-019-0120-7)
Supplement: Supplementary file 1 — Supplementary Note 1: Pulse Dynamics [file 41377_2019_120_MOESM1_ESM.pdf]

Spatiotemporal Continuum Generation in Polariton Waveguides
Supplementary Note 1: Pulse Dynamics

Paul M. Walker,[1,*] Charles E. Whittaker,[1] Dmitry V. Skryabin,[2,3] Emiliano Cancellieri,[1] Ben Royall,[1] Maksym Sich,[1] Ian Farrer,[4] David A. Ritchie,[5] Maurice S. Skolnick,[1] and Dmitry N. Krizhanovskii[1]

[1]*Department of Physics and Astronomy, University of Sheffield, S3 7RH Sheffield, UK*
[2]*Department of Physics, University of Bath, BA2 7AY Bath, UK*
[3]*ITMO University, Kronverksky Avenue 49, St. Petersburg 197101, Russia*
[4]*Department of Electronic and Electrical Engineering, University of Sheffield, S3 7HQ Sheffield, UK*
[5]*Cavendish Laboratory, University of Cambridge, CB3 0HE Cambridge, UK*

PULSE TEMPORAL DYNAMICS

Figures 3(e-h) in the main text show that an initial picosecond pulse breaks into a train of shorter sub-picosecond pulses at elevated powers. The figures show the full wavepacket intensity vs. both time t and transverse position x at the waveguide output at $z = 600\,\mu$m. To better illustrate the gradual breakup of the pulse as it propagates Supplementary figure S1(a) shows the intensity vs. time taken at $x = 0$ and for several different propagation positions z before, as well as at, the waveguide output. The input pulse power $P = 13.4P_0$ and central frequency detuning $\hbar\delta_0 = -6.1$ meV correspond to the data in Figure 3(h) in the main text. It can be seen that over the first 100 µm of propagation the leading edge of the pulse becomes steeper before the pulse splits into a train of shorter pulses with further propagation (increasing z). These shorter pulses then gradually separate from each other. We will now examine this dynamics of steepening and and pulse-splitting in more detail.

Self-Steepening

Supplementary figure S1(b) shows the instantaneous frequency $\omega_i(t) = \partial\phi(t)/\partial t$ for the shorter propagation distances in (a). Here $\phi(t)$ is the phase angle of the lower polariton field as a function of time. As expected, before breakup (e.g. at $z = 100\,\mu$m) the pulse becomes strongly chirped due to self-phase-modulation (SPM) (see 'Methods - Waveguide Nonlinear Properties' in the main text). It can be seen that the sharp leading edge in panel (a) corresponds to higher frequency components, consistent with the defocussing nonlinearity in this system. The edge which corresponds to higher frequency components is the one that sharpens. This is consistent with the experimental observation (Figure 2(h,j) in the main text) that the higher-frequency side of the spectrum was more spectrally spread out since a sharper leading edge corresponds to a greater spread in the frequency domain. Thus both the steepening and the enhanced spectral-spreading on the high-frequency side can be explained by the fact that the nonlinearity arising from polariton-polariton interactions is stronger for the higher frequencies which are closer to the exciton resonance and for which the polaritons have a higher excitonic fraction. We will confirm this later when we compare the simulations to equivalent ones with a frequency-independent nonlinearity.

This steepening is similar to the self-steepening [33] effect which occurs due to higher order (than cubic) nonlinear terms in some materials with a photon-only nonlinearity. In that case the group velocity becomes dependent on intensity so that the peak of a pulse moves towards one edge causing that edge to steepen, just as we observe here. These results show that a self-steepening-like effect exists in the polariton system which behaves in very similar way to that in weakly coupled materials, but much more strongly, and is fundamentally related to the mixed photon-exciton nature of polaritons.

Counterintuitively, the experimentally deduced self-steepening is larger for δ_0 further from the exciton where the system is more photon-like and higher order (frequency dependent) terms in the nonlinearity are expected to be weaker. This can be seen from the experimental spectra (Figure 2 panels (h) and (j) in the main text) where, as noted earlier, the asymmetric spectral spreading is larger for δ_0=-10.5 meV. This is also the case in the simulations. This can be explained because the group velocity dispersion (GVD), which is known to arrest self-steepening [33], also increases strongly as the frequency approaches the exciton. Thus the GVD reduces the asymmetric spreading of the spectrum at smaller detunings.

Pulse Breakup

Soon after the sharp leading edge develops the pulse breaks up into a train of narrower pulses. Temporal compression of pulses into narrower pulses is a very general phenomenon and expected when the signs of nonlinearity and GVD are opposite, as we have here [33]. However, in the polariton system both the GVD and nonlinearity are highly frequency dependent so that generation of a single compressed pulse is not necessarily expected. Supplementary figure S1(c) shows the instantaneous frequency at the positions of the peaks for four of the propagation lengths. It can be seen that immediately after

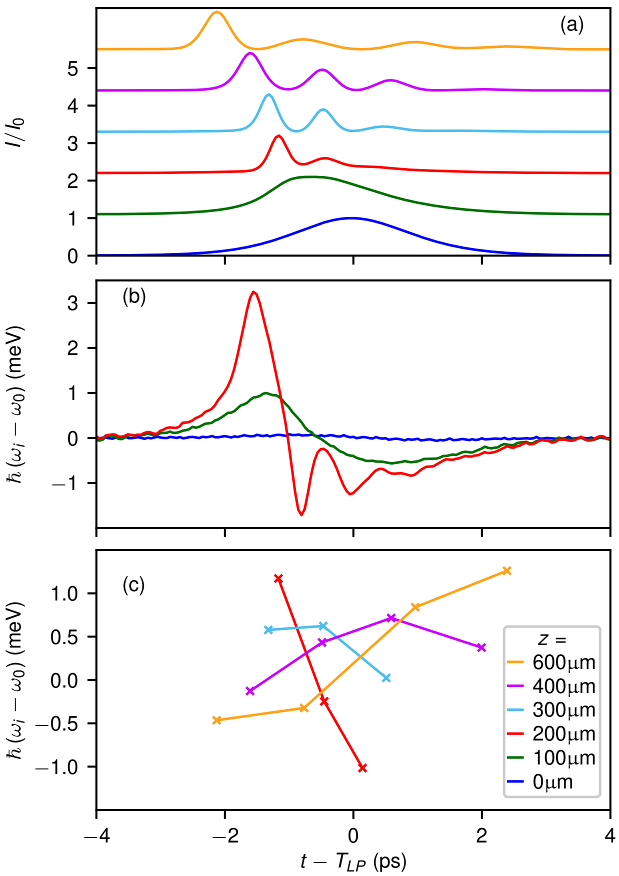

Supplementary Figure S1. Pulse train properties. (a) Fixed-(x, z) Intensity vs. time profiles of the wavepacket at several propagation positions z given in the legend in panel (c) and at the center of the wavepacket ($x = 0$) in the transverse direction (refer to schematic in Figure 1 in the main text). The pulse power and detuning δ_0 are the same as in Figure 3(h) in the main text. (b) Instantaneous frequency vs. time corresponding to the curves in panel (a) for the three shortest propagation lengths, z =100 µm, 200 µm and 300 µm. (c) Instantaneous frequencies at the peaks of the distinct pulses visible in panel (a) for the four longest propagation lengths z =200 µm, 300 µm, 400 µm and 600 µm. $T_{\rm LP}$ is the propagation time over distance z using the polariton group velocity at the pump frequency, defined in 'Methods – Waveguide Linear Properties'.

the breakup into distinct pulses (z=200 µm) the leading pulses correspond to higher frequencies while the trailing pulses are those at lower frequency, in accordance with the chirp imposed by SPM. After some propagation the situation reverses and the high-frequency pulses arrive later than the low frequency ones. This can be explained by the very strong normal GVD induced by the light-matter coupling which causes the more photonic low-frequency pulses to travel faster (see the slope of the dispersion in Figure 1(b) in the main text).

Thus we see that the pulse breakup mechanism proceeds in the following manner, a strong chirp develops due to SPM followed by breakup under the combined influence of nonlinearity and GVD, followed by separation due to the GVD. This happens over a short length scale and for low power and initially narrowband (picosecond) pulse due to the exceptionally strong GVD and nonlinearity in polariton systems. It is important to note that it has previously been shown [15, 42] that one-dimensional pulses in the polariton waveguide system may propagate as bright temporal solitons. In that case the spectrally inhomogeneous nonlinearity and GVD balance each other resulting in strongly chirped soliton pulses. Thus the pulse breakup we observe is only expected for pulses with nonlinear length much shorter than the dispersion length, which is the case here. These may be viewed as initially high-order solitons, or breathers, which are rapidly destabilised by the GVD or frequency dependent nonlinearity, or both [45]. If the pulses were confined in the transverse direction, for example by etching a ridge waveguide profile, one could expect the individual pulses to propagate as separate solitons with different central frequencies and velocities. This can explain why the pulse compresses into a train of solitons at different central frequencies and positions rather than a single narrow peak. We note that in these simulations pulse-breakup still occurs when diffraction is suppressed so that the pulse breakup is not caused by the spatial degree of freedom.

Space-Time coupling through polariton-polariton interactions

The resonant coupling between photon and exciton also has a strong impact on the spatial dynamics of the pulses and the coupling of the spatial and temporal degrees of freedom. In Figure 3(h) in the main text secondary intensity peaks can be seen towards the outer edges of each curved wavefront. These are examined in more detail in Figure 3(k) in the main text, which shows the spatio-temporal pulse envelopes at two positions in the waveguide for a slightly higher input power. It can be seen that with increasing z the outer edges of the pulse train gradually separate into a secondary wavefront with opposite curvature to the main train. This secondary wavefront propagates more slowly than and thus separates from the main train. We examine the origin of this radiation in more detail in Supplementary figure S2. Panel (a) shows the instantaneous transverse spatial frequency $k_{\rm i}$ in the vicinity the curved wavefronts of Figure 3(h) in the main text. $k_{\rm i}$ is higher at large x which is the signature of the spatial self-defocussing we expect for our repulsive nonlinearity [27]. In fact this defocussing is the due to SPM in the spatial coordinate combined with diffraction [45]. Supplementary figure S2(b) shows the corresponding instantaneous frequency $\omega_{\rm i}$ relative to the pump. The higher frequency components are also located towards the outer edges of the wavefronts at high

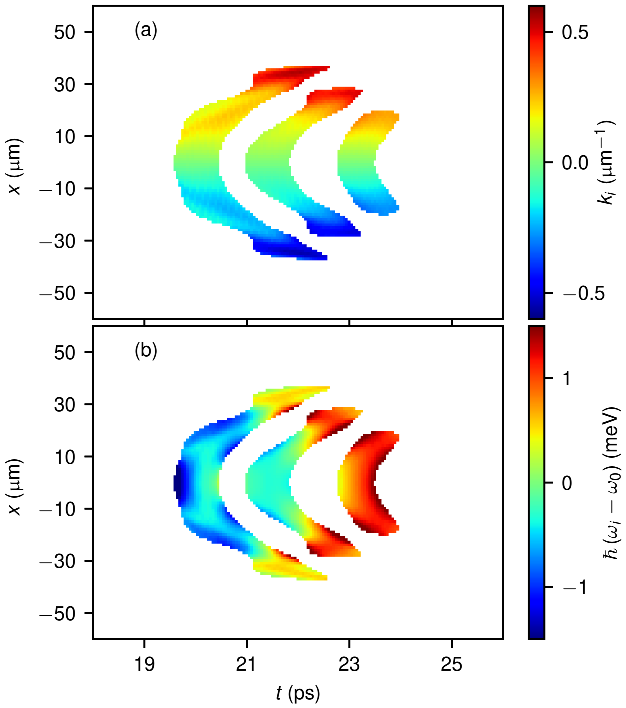

Supplementary Figure S2. Instantaneous spatial frequency k_i (a) and frequency minus pump frequency (b) of the wavefronts in Figure 3(h) in the main text. Data is shown for intensities greater than 0.1 of the peak intensity of the wavefronts.

x which means that the frequencies closer to the exciton experience more nonlinear self-defocussing. This is in agreement with the experimental spectra (Fig 2(d) in the main text) showing a greater spread in angular spectrum (and hence more spatial SPM) for wavelengths closer to the exciton. The trailing wavefront in Figure 3(k) in the main text is then composed of the highest frequency and hence most spatially defocussed polaritons, which causes spatial separation. These are also the slowest propagating, which causes it to also separate temporally.

COMPARISON WITH CASE OF SPECTRALLY INDEPENDENT NONLINEARITY

The strong photon-exciton coupling causes both the nonlinearity and dispersion to be strongly dependent on frequency. To investigate how much the frequency dependent nonlinearity contributes to the evolution we here present simulations where the polariton nonlinearity has been made independent of frequency. The system of equations used to simulate this case is given in Equations S1.

$$i\frac{\partial u}{\partial t} = \left[\delta_{\text{lin}}\left(Q_z, k_x\right) + \delta_{\text{NL}}\left(|u|^2\right)\right] u \qquad \text{(S1a)}$$

$$\delta_{\text{lin}}\left(Q_z, k_x\right) = \frac{1}{2}\left(\delta_{\text{p}} - i\gamma_{\text{e}} - \sqrt{\left(\delta_{\text{p}} + i\gamma_{\text{e}}\right)^2 + \Omega^2}\right) \qquad \text{(S1b)}$$

$$\delta_{\text{p}} = v_{\text{g}}\left(Q_z + \frac{k_x^2}{2k_{\text{e}}}\right) - i\gamma_{\text{p}} \qquad \text{(S1c)}$$

Here u is the lower polariton field, γ_{e} and γ_{p} are the exciton and photon loss rates, Q_z and k_x are the longitudinal and transverse momenta, Ω is the Rabi splitting and v_{g} is the photon group velocity, all as defined in the main text. The equations were solved using the same numerical method as the coupled photon and exciton field equations (Equations 1(a) and 1(b) in the main text). The linear evolution term $\delta_{\text{lin}}\left(Q_z, k_x\right)$, solved in the (spatial) frequency domain, captures the polariton dispersion and frequency-dependent loss rate. The nonlinear term $\delta_{\text{NL}}\left(|u|^2\right)$ was obtained by solving Equations 1(a) and 1(b) in the main text for the case of a continuous wave at a fixed momentum, for which they can be reduced to a fourth order polynomial in the polariton frequency. Solving this for the nonlinear and linear (e.g. with $g = 0$) cases and taking the difference gives the nonlinear contribution to the polariton evolution. This solution is frequency independent but accurately captures the saturating nature of the polariton nonlinearity. The population of the upper polariton was found to be negligible and so we consider only the lower polariton field here. The strength of the nonlinearity was calibrated by ensuring that the same peak nonlinear self-phase was accumulated over the first 5 micrometers of pulse propagation in both the full and frequency-independent cases. During this early evolution the pulse intensity profile did not vary significantly.

Self-Steepening

We first consider whether the self-steepening effect is still present for the frequency-independent nonlinearity. Supplementary figures S3(a) and (b) show the pulse intensity profiles at a range of positions z in the waveguide for the full and frequency-independent cases respectively. It can be seen in panel (a) that the leading edge of the pulse gradually sharpens with increasing propagation distance z so that the peak of the pulse becomes markedly to one side of its center of mass. By contrast, in the frequency-independent case in panel (b) the pulse narrows symmetrically. Thus the self-steepening effect discussed above arises from the frequency-dependence of the polariton nonlinearity. We note that in both panels (a) and (b) at the largest z a secondary pulse begins to split off on the right hand side of the peak. Thus, although

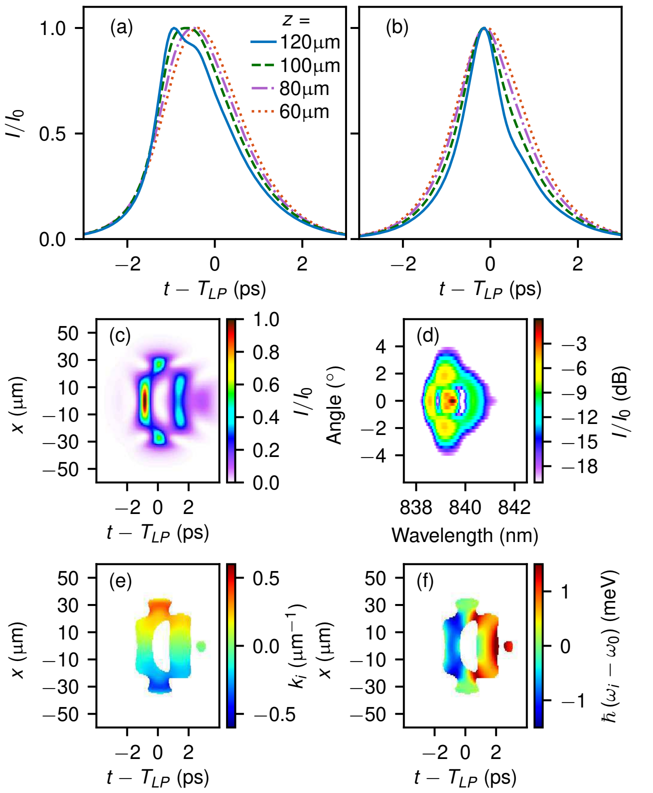

Supplementary Figure S3. (a,b) Sections of the wavepacket envelope through $x = 0$ at several z for input conditions corresponding to Figure 3(h) in the main text for the full (a) and frequency-independent (b) cases. (c) Spatio-temporal envelope of the pulse at z=600 µm for input conditions as in panel (b). (d) Spectrum corresponding to panel (c). (e) Instantaneous spatial frequency k_i and (f) instantaneous frequency ω_i minus pump frequency ω_0 for intensities greater than 0.1 of the peak. T_{LP} is the propagation time over distance z using the polariton group velocity at the pump frequency, defined in 'Methods – Waveguide Linear Properties'.

the self-steepening results from the frequency-dependent nonlinearity, the pulse-splitting can occur without it. We will discuss this in more detail later in this section.

Space-Time Coupling

In Supplementary figure S3(c-f) we show respectively the pulse intensity, spectrum, instantaneous transverse wavevector and instantaneous frequency. Supplementary figures S3(c) and (d) are the frequency-independent equivalents of the main-text figures 3(h) and 3(d) respectively. It can be seen by comparing Supplementary figure S3(c) and Figure 3(h) in the main text that the wavefronts are markedly less curved in the frequency-independent case, especially in the central region near $x = 0$. Towards the edges of the wavepacket some curvature can still be seen. Similarly, by comparing Supplementary figure S3(d) and Figure 3(d) in the main text it can be seen that the spectrum is mores symmetrical and less triangular in shape, with the degree of angular broadening being less dependent on frequency. However the spectrum is not perfectly symmetrical around any particular wavelength. Thus the frequency-dependence of the polariton nonlinearity contributes strongly to the curvature of the wavefronts after pulse breakup and also to the characteristic triangular spectral shape, but is not the only contribution. We note that over propagation distances which are long compared to the nonlinear and disperison lengths, such that the strong pulse modulation we show in Supplementary figures S3(c-f) is possible, the effects of nonlinearity and dispersion interact with each other and cannot be disentangled. Thus although we have removed the frequency dependence of the nonlinearity from our equations the solution may still, to a lesser extent, exhibit similar features to the full case because of the strong polariton dispersion, which we have retained.

Supplementary figures S3(e) and (f) are the frequency-independent equivalents of Supplementary figures S2 (a) and (b) respectively. Comparing Supplementary figure S3(e) and Supplementary figure S2(a) it can be seen that in both cases the instantaneous transverse spatial frequency k_{i} is higher at large x so that the pulse is self-defocussing as expected. In discussing Supplementary figure S2(b) earlier we noted that the higher ω_i frequency components are also located towards the outer edges of the wavefronts, at high x, so that the slower-propagating frequencies closer to the exciton experience more defocussing. This effect is much less pronounced in Supplementary figure S3(f) where the instantaneous frequency ω_i is only weakly dependent on x by comparison. This explains why the wavefronts curve much less during propagation in the case of frequency-independent nonlinearity.

Pulse Breakup

In Supplementary figure S4 we present the equivalent data to that in Supplementary figure S1 but for the case of frequency-independent nonlinearity. It can be seen from Supplementary figure S4(a) that the pulse still breaks up. We note that in the case of the basic Nonlinear Schrodinger Equation with perturbations, as discussed in Reference 45, the break-up of a high-energy pulse into a train can be brought on by a wide range of perturbations which can include frequency dependence of the nonlinearity or higher-order dispersion terms. In the case studied here we always have strong high order dispersive terms even in the case of the frequency-independent nonlinearity. Thus it appears that pulse breakup in the polariton system can be caused by the strong dispersion which originiates in the photon-exciton coupling. The frequency-dependent nonlinearity may also contribute,

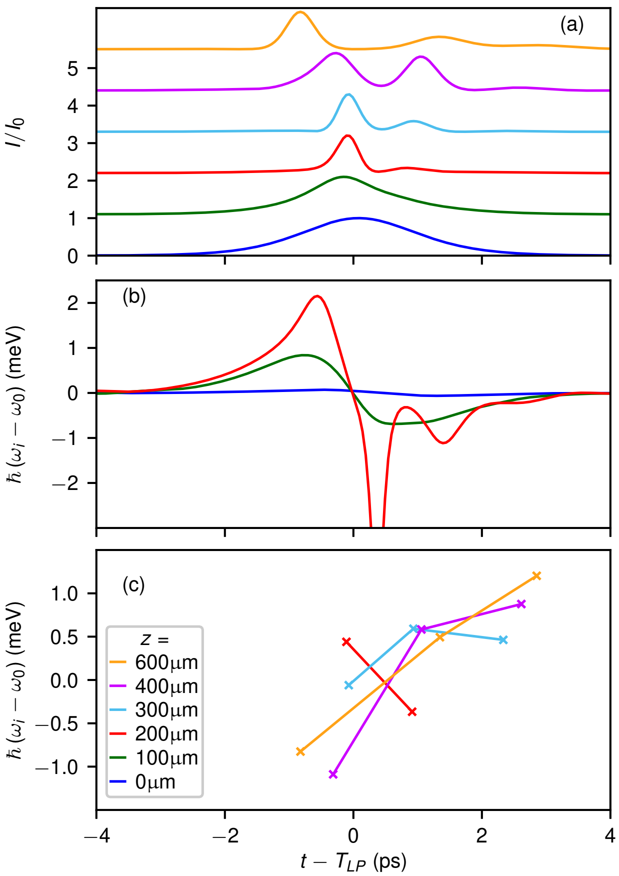

Supplementary Figure S4. Pulse train properties for the case of frequency independent nonlinearity. (a) Sections of the wavepacket envelope through $x = 0$ at several z (see legend in panel (b)) for input conditions corresponding to Figure 3(h) in the main text. (b) Instantaneous frequency vs. time corresponding to panel (a) for three different z. (c) Instantaneous frequencies at the peaks of the distinct pulses visible in (a). $T_{\rm LP}$ is the propagation time over distance z using the polariton group velocity at the pump frequency, defined in 'Methods – Waveguide Linear Properties'.

but it is difficult to separate the two effects. Comparing all the panels in Supplementary figure S1 and Supplementary figure S4 it can be seen that the breakup mechanism is very similar in both cases. First there is a strong chirp generated by SPM so that higher frequency components are located at the front of the pulse. Then the pulse breaks up, with the higher frequency sub-pulses still located at the leading edge. At long propagation distances, the strong dispersion begins to dominate and the lower frequency pulses overtake the higher frequency ones.

SUMMARY

In summary, the main effects of the frequency-dependent nonlinearity are (1) determining the particular shape of the initial chirp which then effects the central frequencies of the pulses after breakup, (2) causing a steepening of the leading edge of pulses at short propagation distances before the break-up, and (3) driving higher frequency components out to larger transverse momentum k_x and position x thus creating stronger correlations between the spatial and temporal degrees of freedom. These latter manifest as a triangular spectral shape and curved wavefront shape. Even without the spectrally-dependent nonlinearity, however, the polaritonic nature of the system strongly influences the spatio-temporal dynamics via the strong high-order dispersion caused by the anti-crossing of the photon and exciton modes. We note that we also performed simulations for the case $\delta_{\rm NL} = g_{LP}\,|u|^2$, e.g. for a simple Kerr-like nonlinearity, which does not saturate in the same way as the polariton nonlinearity, and obtained qualitatively the same results.
