# Supplementary material for: Spatiotemporal continuum generation in polariton waveguides
Source: Light Sci Appl. 2019 Jan 16;8:6. doi: 10.1038/s41377-019-0120-7 (PMC6333623; doi:10.1038/s41377-019-0120-7)
Supplement: Supplementary file 2 — Supplementary Note 2: Modulation Instability [file 41377_2019_120_MOESM2_ESM.pdf]

Spatiotemporal Continuum Generation in Polariton Waveguides Supplementary Note 2: Modulation Instability

Paul M. Walker,[1,*] Charles E. Whittaker,[1] Dmitry V. Skryabin,[2,3] Emiliano Cancellieri,[1] Ben Royall,[1] Maksym Sich,[1] Ian Farrer,[4] David A. Ritchie,[5] Maurice S. Skolnick,[1] and Dmitry N. Krizhanovskii[1]

[1]*Department of Physics and Astronomy, University of Sheffield, S3 7RH Sheffield, UK*

[2]*Department of Physics, University of Bath, BA2 7AY Bath, UK*

[3]*ITMO University, Kronverksky Avenue 49, St. Petersburg 197101, Russia*

[4]*Department of Electronic and Electrical Engineering, University of Sheffield, S3 7HQ Sheffield, UK*

[5]*Cavendish Laboratory, University of Cambridge, CB3 0HE Cambridge, UK*

I. BASIC EQUATIONS

The state of the system is descibed by complex scalar electric and exciton polarisation fields $E(x, z, t)$ and $P(x, z, t)$ respectively. We work within paraxial and slowly varying envelope approximations with the slowly varying fields $A(x, z, t)$ and $\Psi(x, z, t)$ such that $E = A\exp(ik_\mathrm{e}z - i\omega_\mathrm{e}t)$, $P = \Psi\exp(ik_\mathrm{e}z - i\omega_\mathrm{e}t)$. The evolution equations for the system are then given by the coupled equations (1).

$$\left(i\frac{\partial}{\partial t} + i\gamma_\mathrm{p} + v_\mathrm{g}\left(i\frac{\partial}{\partial z} + \frac{1}{2k_\mathrm{e}}\frac{\partial^2}{\partial x^2}\right)\right)A = \left(\frac{\Omega}{2}\right)\Psi \tag{1a}$$

$$\left(i\frac{\partial}{\partial t} + i\gamma_\mathrm{e} - g\left|\Psi\right|^2\right)\Psi = \left(\frac{\Omega}{2}\right)A \tag{1b}$$

The variables ω_e, k_e, v_g, γ_p, γ_e, Ω, and g are all real and positive. They respectively describe the exciton frequency, the photon wavenumber and group velocity at ω_e, the photon and exciton loss rates, the strength of light matter coupling and the nonlinear renormalisa-

* p.m.walker@sheffield.ac.uk

tion of the exciton frequency per unit exciton density. The variables A and Ψ are in general complex.

II. CW SOLUTION

We insert the ansatz

$$A = A_0(z)\exp\left(i(k_\mathrm{p}x - \delta t)\right) \tag{2a}$$

$$\Psi = \Psi_0(z)\exp\left(i(k_\mathrm{p}x - \delta t)\right) \tag{2b}$$

Here k_p, δ are taken to be real and $A_0(z)$ and $\Psi_0(z)$ are complex functions of z only. Inserting Equations (2) into Equation (1b) gives

$$A_0 = \left(\frac{\delta + i\gamma_\mathrm{e} - g\left|\Psi_0\right|^2}{(\Omega/2)}\right)\Psi_0 \tag{3}$$

Thus we have a relation giving the photon field for a given exciton field. Inserting Equations (2) and Equation (3) into Equation (1a) also gives

$$iv_\mathrm{g}\frac{\partial A_0}{\partial z} + \left(\delta + i\gamma_\mathrm{p} - \frac{(\Omega/2)^2}{\delta + i\gamma_\mathrm{e} - g\left|\Psi_0\right|^2} - \frac{v_\mathrm{g}k_\mathrm{p}^2}{2k_\mathrm{e}}\right)A_0 = 0 \tag{4}$$

The value of $\left|\Psi_0\right|^2$ needed in Equation (4) may be obtained from A_0 by taking the square modulus of both sides of Equation (3) and solving the resulting cubic algebraic equation (see the supplementary material of Reference 27 for more details). Then Equation (4) may be solved numerically using e.g. a Runge-Kutta method.

In the lossless case $\gamma_\mathrm{p} = \gamma_\mathrm{e} = 0$ it can be shown that $\left|A_0\right|^2$ and hence $\left|\Psi_0\right|^2$ are independent of z (using $\partial(A_0{A_0}^*)/\partial z = A_0(\partial {A_0}^*/\partial z) + {A_0}^*(\partial A_0/\partial z)$, Equation (4), and its conjugate). Then Equations (3) and (4) are simply solved by $A_0(z) = A_0(0)\exp\left(iQ_z z\right)$ where Q_z is given by

$$Q_z = \frac{1}{v_\mathrm{g}}\left(\delta - \frac{(\Omega/2)^2}{\delta - g\left|\Psi_0\right|^2} - \frac{v_\mathrm{g}k_\mathrm{p}^2}{2k_\mathrm{e}}\right) \tag{5}$$

III. EVOLUTION EQUATIONS FOR PERTURBATIONS

We now introduce perturbations $a(x,z,t)$ and $\psi(x,z,t)$ which are taken to be much smaller in amplitude than A_0 and Ψ_0. The new ansatz for the fields is

$$A(x,z,t) = \frac{A_0(z)}{A_0(0)} e^{i(k_\mathrm{p} x - \delta t)} \left(A_0(0) + a(x,z,t) \right) \tag{6a}$$

$$\Psi(x,z,t) = \frac{\Psi_0(z)}{\Psi_0(0)} e^{i(k_\mathrm{p} x - \delta t)} \left(\Psi_0(0) + \psi(x,z,t) \right) \tag{6b}$$

Inserting this ansatz into Equations (1) and retaining only terms linear in (or independent of) a and ψ (because they are small) we obtain evolution equations for the perturbations

$$i\frac{\partial a}{\partial t} + v_\mathrm{g}\left(i\frac{\partial}{\partial z} + i\frac{k_\mathrm{p}}{k_\mathrm{e}}\frac{\partial}{\partial x} + \frac{1}{2k_\mathrm{e}}\frac{\partial^2}{\partial x^2} \right) a + \left(\frac{(\Omega/2)^2}{\delta_\mathrm{R}(z) + i\gamma_\mathrm{e}} \right) a = \left(1/\sigma(z)\right)\left(\Omega/2\right)\psi \tag{7a}$$

$$i\frac{\partial \psi}{\partial t} + \left(\delta_\mathrm{R} + i\gamma_\mathrm{e}\right)\psi - \omega_\mathrm{NL}(z)\left(\psi + \psi^*\right) = \sigma(z)\left(\Omega/2\right) a \tag{7b}$$

$$\sigma(z) = \left(\delta_\mathrm{R}(z) + i\gamma_\mathrm{e}\right) / \left(\delta_\mathrm{R}(0) + i\gamma_\mathrm{e}\right) \tag{7c}$$

Here $\omega_\mathrm{NL}(z) = g\left|\Psi_0(z)\right|^2$ is the nonlinear renormalisation of the exciton oscillator frequency due to the exciton density and $\delta_\mathrm{R}(z) = \delta - \omega_\mathrm{NL}(z)$ is the renormalised detuning between the exciton and pump frequencies.

In deriving these we made use of Equation (3) to eliminate A_0 from (7b). Equations (4) and (3) were both used (via the useful intermediate relations in Equations (8)) to eliminate A_0 and $\partial A_0/\partial z$ from Equations (7a).

$$\left(\delta + i\gamma_\mathrm{p} - \frac{v_\mathrm{g} k_\mathrm{p}^2}{2k_\mathrm{e}}\right) A_0(0) + i v_\mathrm{g}\left(\frac{A_0(0)}{A_0(z)}\right)\frac{\partial A_0}{\partial z}\Big|_z - \left(\frac{\Omega}{2}\right)\left(\frac{\Psi_0(z)}{\Psi_0(0)}\frac{A_0(0)}{A_0(z)}\right)\Psi_0(0) = 0 \tag{8a}$$

$$i v_\mathrm{g}\frac{\partial A_0}{\partial z}\Big|_z = -\left(\delta + i\gamma_\mathrm{p} - \frac{v_\mathrm{g} k_\mathrm{p}^2}{2k_\mathrm{e}}\right) A_0(z) + \left(\Omega/2\right)\Psi_0(z) \tag{8b}$$

$$\left(\frac{A_0(z)}{\Psi_0(z)}\frac{\Psi_0(0)}{A_0(0)}\right) = \sigma(z) \tag{8c}$$

We have, without loss of generality, chosen $\Psi_0(0)$ to be real and thus fixed the reference phase for the system.

IV. INSTABILITY GROWTH RATE

Since Equation (7b) couples the perturbations with their complex conjugates, and hence couples positive and negative frequency components in the Fourier transformed fields, we insert the following ansatz for the perturbations

$$a(x,z,t) = a_{\mathrm{I}-}(z)\exp\left(i(kx-\Delta t)\right) + a_{\mathrm{S}-}(z)^{*}\exp\left(-i(kx-\Delta t)\right) + a_{\mathrm{I}+}(z)\exp\left(-i(kx+\Delta t)\right) + a_{\mathrm{S}+}(z)^{*}\exp\left(i(kx+\Delta t)\right) \tag{9a}$$

$$\psi(x,z,t) = \psi_{\mathrm{I}-}(z)\exp\left(i(kx-\Delta t)\right) + \psi_{\mathrm{S}-}(z)^{*}\exp\left(-i(kx-\Delta t)\right) + \psi_{\mathrm{I}+}(z)\exp\left(-i(kx+\Delta t)\right) + \psi_{\mathrm{S}+}(z)^{*}\exp\left(i(kx+\Delta t)\right) \tag{9b}$$

These correspond to perturbations which are transverse plane waves and monochromatic (PM). The offsets of the perturbation transverse wavenumbers and frequencies from those of the pump have magnitudes of k and Δ. We take k and Δ to be positive real values and, as can be seen, the perturbation expressions sum over all the possible positive and negative combinations.

Substituting this ansatz into the two evolution equations Equations (7) and collecting coefficients of $\exp\left(\pm i(kx \pm \Delta t)\right)$ leads to eight equations. There are four equations involving $a_{\mathrm{I}-}$, $a_{\mathrm{S}-}$, $\psi_{\mathrm{I}-}$, $\psi_{\mathrm{S}-}$ and a separate four equations for the coefficients with '+' in the subscripts. These two sets are uncoupled and identical apart from the substitution $k \to -k$ and so they may be solved independently. In the following we will consider only the set for the '$-$' coefficients and will drop the '$-$' part of the subscript. The four equations are given in

$$\left(v_{\mathrm{g}} q_{\mathrm{L}} - \Delta + \omega_{\mathrm{T}} - \eta_0(z)\left(\delta_{\mathrm{R}}(z) - i\gamma_{\mathrm{e}}\right)\right) a_{\mathrm{I}} + \frac{\Omega}{2\sigma(z)}\psi_{\mathrm{I}} = 0 \tag{10a}$$

$$\left(\Delta - v_{\mathrm{g}} q_{\mathrm{L}} + \omega_{\mathrm{T}} - \eta_0(z)\left(\delta_{\mathrm{R}}(z) + i\gamma_{\mathrm{e}}\right)\right) a_{\mathrm{S}} + \frac{\Omega}{2\sigma^{*}(z)}\psi_{\mathrm{S}} = 0 \tag{10b}$$

$$\left[\omega_{\mathrm{NL}}(z) - \delta_{\mathrm{R}}(z) - (i\gamma_{\mathrm{e}} + \Delta)\right]\psi_{\mathrm{I}} + \omega_{\mathrm{NL}}(z)\psi_{\mathrm{S}} + \frac{\sigma(z)\Omega}{2} a_{\mathrm{I}} = 0 \tag{10c}$$

$$\left[\omega_{\mathrm{NL}}(z) - \delta_{\mathrm{R}}(z) + (i\gamma_{\mathrm{e}} + \Delta)\right]\psi_{\mathrm{S}} + \omega_{\mathrm{NL}}(z)\psi_{\mathrm{I}} + \frac{\sigma^{*}(z)\Omega}{2} a_{\mathrm{S}} = 0 \tag{10d}$$

We have made use of the following definitions for the sake of brevity

$$q_{\mathrm{L}} = -i\partial/\partial z + k_{\mathrm{p}} k / k_{\mathrm{e}} \tag{11a}$$

$$\omega_{\mathrm{T}} = v_{\mathrm{g}} k^2 / (2k_{\mathrm{e}}) \tag{11b}$$

$$\eta_0(z) = (\Omega/2)^2 / \left(\delta_{\mathrm{R}}(z)^2 + \gamma_{\mathrm{e}}^2\right) = \left|\Psi_0\right|^2 / \left|A_0\right|^2 \tag{11c}$$

Note also that we have taken the complex conjugate of the equations coming from collecting the coefficients of terms containing $\exp\left(+i\Delta t\right)$.

The algebraic equations (10c) and (10d) may be used to eliminate the exciton fields.

$$\psi_{\mathrm{S}} = \left(\frac{\Omega}{2}\right) \frac{\left(\delta_{\mathrm{R}} - \omega_{\mathrm{NL}} + (\Delta + i\gamma_{\mathrm{e}})\right) \sigma^* a_{\mathrm{S}} + \omega_{\mathrm{NL}} \sigma a_{\mathrm{I}}}{\left(\delta_{\mathrm{R}} - (\Delta + i\gamma_{\mathrm{e}})\right)\left(\delta_{\mathrm{R}} + (\Delta + i\gamma_{\mathrm{e}})\right) - 2\omega_{\mathrm{NL}}\delta_{\mathrm{R}}} \tag{12a}$$

$$\psi_{\mathrm{I}} = \left(\frac{\Omega}{2}\right) \frac{\left(\delta_{\mathrm{R}} - \omega_{\mathrm{NL}} - (\Delta + i\gamma_{\mathrm{e}})\right) \sigma a_{\mathrm{I}} + \omega_{\mathrm{NL}} \sigma^* a_{\mathrm{S}}}{\left(\delta_{\mathrm{R}} - (\Delta + i\gamma_{\mathrm{e}})\right)\left(\delta_{\mathrm{R}} + (\Delta + i\gamma_{\mathrm{e}})\right) - 2\omega_{\mathrm{NL}}\delta_{\mathrm{R}}} \tag{12b}$$

We then obtain a Hamiltonian describing the evolution of the perturbations $(a_{\mathrm{I}}, a_{\mathrm{S}})$.

$$\begin{aligned} &\left[-iv_{\mathrm{g}}\frac{\partial}{\partial z} - \left(\Delta\left(1+\eta\right) + i\left(\eta - \eta_0\right)\gamma_{\mathrm{e}} - \frac{v_{\mathrm{g}} k_{\mathrm{p}} k}{k_{\mathrm{e}}}\right)\right] \begin{pmatrix} a_{\mathrm{I}} \\ a_{\mathrm{S}} \end{pmatrix} = \\ &\begin{pmatrix} -\left[\left(\eta - \eta_0\right)\delta_{\mathrm{R}} + \omega_{\mathrm{T}} - \eta\omega_{\mathrm{NL}}\right] & -\eta\omega_{\mathrm{NL}}\frac{\sigma^*}{\sigma} \\ \eta\omega_{\mathrm{NL}}\frac{\sigma}{\sigma^*} & \left[\left(\eta - \eta_0\right)\delta_{\mathrm{R}} + \omega_{\mathrm{T}} - \eta\omega_{\mathrm{NL}}\right] \end{pmatrix} \begin{pmatrix} a_{\mathrm{I}} \\ a_{\mathrm{S}} \end{pmatrix} \end{aligned} \tag{13}$$

Here we have defined

$$\eta(z) = \frac{\left(\Omega/2\right)^2}{\left[\delta_{\mathrm{R}} - (\Delta + i\gamma_{\mathrm{e}})\right]\left[\delta_{\mathrm{R}} + (\Delta + i\gamma_{\mathrm{e}})\right] - 2\omega_{\mathrm{NL}}\delta_{\mathrm{R}}} \tag{14}$$

We note that the coefficients of the Hamiltonian are functions of z (although not of $a_{\mathrm{I}}, a_{\mathrm{S}}$) and it must in general be solved numerically. However, provided that the losses are sufficiently small so that we can consider evolution of the perturbations over a length small compared to the loss length, the matrix can be approximated as constant with z. Then the system can be solved by diagonalizing the matrix.

$$\frac{\partial}{\partial z}\begin{pmatrix} u_1 \\ u_2 \end{pmatrix} = \begin{pmatrix} i\left(q_1 - q_2\right) & 0 \\ 0 & i\left(q_1 + q_2\right) \end{pmatrix}\begin{pmatrix} u_1 \\ u_2 \end{pmatrix} \tag{15a}$$

$$v_{\mathrm{g}} q_1 = \Delta\left(1+\eta\right) + i\left(\eta - \eta_0\right)\gamma_{\mathrm{e}} - \frac{v_{\mathrm{g}} k_{\mathrm{p}} k}{k_{\mathrm{e}}} \tag{15b}$$

$$v_{\mathrm{g}} q_2 = \sqrt{\omega_{\mathrm{T}}^2 + 2\omega_{\mathrm{T}}\left[\left(\eta-\eta_0\right)\delta_{\mathrm{R}} - \eta\omega_{\mathrm{NL}}\right] + \delta_{\mathrm{R}}\left(\eta-\eta_0\right)\left[\left(\eta-\eta_0\right)\delta_{\mathrm{R}} - 2\eta\omega_{\mathrm{NL}}\right]} \tag{15c}$$

$$\begin{pmatrix} u_1 \\ u_2 \end{pmatrix} = \frac{1}{2}\begin{pmatrix} -\frac{\sigma}{\sigma^*}\frac{\eta\omega_{\mathrm{NL}}}{v_{\mathrm{g}} q_2} & \left(1 - \sqrt{1 + \left(\frac{\eta\omega_{\mathrm{NL}}}{v_{\mathrm{g}} q_2}\right)^2}\right) \\ \frac{\sigma}{\sigma^*}\frac{\eta\omega_{\mathrm{NL}}}{v_{\mathrm{g}} q_2} & \left(1 + \sqrt{1 + \left(\frac{\eta\omega_{\mathrm{NL}}}{v_{\mathrm{g}} q_2}\right)^2}\right) \end{pmatrix}\begin{pmatrix} a_{\mathrm{I}} \\ a_{\mathrm{S}} \end{pmatrix} \tag{15d}$$

$$\left(\eta-\eta_0\right)\delta_{\mathrm{R}} - m\eta\omega_{\mathrm{NL}} = \eta\left(\frac{\delta_{\mathrm{R}}^2}{\delta_{\mathrm{R}}^2 + \gamma_{\mathrm{e}}^2}\right)\left[\frac{\Delta^2}{\delta_{\mathrm{R}}} + (2-m)\omega_{\mathrm{NL}} + \frac{2i\Delta\gamma_{\mathrm{e}}}{\delta_{\mathrm{R}}} - \frac{m\gamma_{\mathrm{e}}^2\omega_{\mathrm{NL}}}{\delta_{\mathrm{R}}^2}\right] \tag{15e}$$

The physical interpretation of ω_{T} is the kinetic energy associated with transverse wavenumber relative to the pump. Meanwhile $1+\eta_0$ may be interpreted as an effective group index which reduces the polariton velocity at the pump frequency relative to the uncoupled photon velocity and $1+\eta$ is the same but averaged over signal and idler states on the polariton branches, renormalized by the pump density. Equivalently, they are related to the ratios of exciton and photon densities.

The gain for the two solutions is then given by $G = -2\Im\left(q_1 \pm q_2\right)$. We will now consider the form of the gain in more detail. To better obtain physical insight we will consider the lossless case. Then $v_{\mathrm{g}} q_1$ is real while $v_{\mathrm{g}} q_2$ is either purely real or imaginary. In that case only the u_1 perturbation can grow exponentially. The gain is then given by Equation (16).

$$G = \frac{2}{v_{\mathrm{g}}}\left[\Delta^2\eta\left(\eta_0 - \eta\right) + 2\eta\left(\frac{\Delta^2}{g\Psi_0^2 - \delta} - g\Psi_0^2\right)\omega_{\mathrm{T}} - \omega_{\mathrm{T}}^2\right]^{1/2} \tag{16}$$

For a detuning less than some critical value, $\Delta^2 \leq \Delta_{\mathrm{s}}^2$, (see Equation (17)) the peak gain occurs at $k=0$ e.g. for waves travelling with the same transverse momentum as the pump.

$$\Delta_{\mathrm{s}}^2 = g\Psi_0^2\left(g\Psi_0^2 - \delta\right) \tag{17}$$

When $k=0$ then there is gain provided that $\Delta^2 < 2\Delta_{\mathrm{s}}^2$. The peak gain occurs at $\Delta_{\mathrm{m,k=0}}^2$ and has value $G_{\mathrm{m,k=0}}$ given by Equations (18a) and (18b) respectively.

$$\Delta^2_{\mathrm{m,k=0}} = \Delta^2_{\mathrm{s}} \left(3g\Psi_0^2 - \delta\right) / \left(2g\Psi_0^2 - \delta\right) \tag{18a}$$

$$G_{\mathrm{m,k=0}} = \frac{2}{v_{\mathrm{g}}} \left(\frac{g\Psi_0^2}{g\Psi_0^2 - \delta}\right) \sqrt{\frac{1}{\left(g\Psi_0^2 - \delta\right)\left(3g\Psi_0^2 - \delta\right)}} \tag{18b}$$

For $\Delta^2 > \Delta^2_{\mathrm{s}}$ the peak gain occurs at $k = k_{\mathrm{m}}$ defined below.

$$k_{\mathrm{m}} = \left[\frac{2k_{\mathrm{e}}}{v_{\mathrm{g}}} \frac{\eta}{\left(g\Psi_0^2 - \delta\right)} \left(\Delta^2 - \Delta^2_{\mathrm{s}}\right)\right]^{1/2} \tag{19}$$

It is interesting to note that $\Delta^2_{\mathrm{m,k=0}}$ is always larger than Δ^2_{s} so that the peak gain along k=0 is always lower than the gain at some finite k for the same perturbation frequency.

Finally we note that assuming a pump on the lower polariton branch ($\delta < 0$) then for large perturbation detunings $\Delta^2 > \left(g\Psi_0^2 - \delta\right)\left(3g\Psi_0^2 - \delta\right)$ there is never any gain since η becomes negative while at the same time $\Delta^2 > \left(g\Psi_0^2 - \delta\right) g\Psi_0^2$ is clearly fulfilled if $\Delta^2 > \left(g\Psi_0^2 - \delta\right)\left(3g\Psi_0^2 - \delta\right)$ so that all three terms in the square root of Equation (16) are guaranteed to be negative.
